# Novel Ultrasonic Pretreatment for Improving Drying Performance and Physicochemical Properties of *Licorice* Slices During Radio Frequency Vacuum Drying

**DOI:** 10.3390/foods13244071

**Published:** 2024-12-17

**Authors:** Jun Li, Fangxin Wan, Xiaopeng Huang, Xiaoping Yang, Zepeng Zang, Yanrui Xu, Bowen Wu, Kaikai Zhang, Guojun Ma

**Affiliations:** College of Mechanical and Electrical Engineering, Gansu Agricultural University, Lanzhou 730070, China; lijun@st.gsau.edu.cn (J.L.);

**Keywords:** radio frequency vacuum drying, ultrasonic pretreatment, *Licorice* slices, drying characteristics, physicochemical quality

## Abstract

To enhance the physicochemical quality, drying efficiency, and nutrient retention of dried *Licorice* products, this study investigated the effects of ultrasonic pretreatment on the radio frequency vacuum (RFV) drying characteristics, microstructure, and retention of natural active substances in *Licorice* slices. The ultrasonic time, power, and frequency were considered as experimental factors. The results showed that, compared with conventional RFV drying, ultrasonic pretreatment reduced the drying time of *Licorice* slices by 20–60 min. The Weibull model accurately described the moisture ratio changes under different pretreatment conditions (R^2^ > 0.9984, χ^2^ < 2.381 × 10^−5^). The optimal retention of polysaccharides, total phenols, total flavonoids, and antioxidants was achieved under pretreatment conditions of 30 min of ultrasonic time, 180 W of ultrasonic power, and 40 kHz of ultrasonic frequency. Furthermore, ultrasonic pretreatment preserved the internal cellular structure of *Licorice* slices, maintaining intact tissue cells and well-defined microchannels. However, a slight reduction in sample color was observed following ultrasound application. In conclusion, ultrasonic pretreatment significantly improved the RFV drying process for *Licorice* slices by enhancing drying efficiency, preserving active ingredients, and optimizing the physicochemical quality of the dried product. This study provides novel insights and methods for optimizing the drying process of *Licorice*, offering a foundation for further research and industrial applications.

## 1. Introduction

*Glycyrrhiza uralensis* Fisch., commonly known as *Licorice*, refers to the dried roots and rhizomes of a perennial leguminous plant in the genus Glycyrrhiza [1,2]. Native to Asia and Europe, it is widely distributed in regions such as China, Mongolia, and Russia. As a traditional medicinal herb in Chinese medicine, *Licorice* exhibits significant pharmacological properties and therapeutic effects, including hepatoprotection, antitumor, and transaminase-lowering activities [3,4]. However, *Licorice* has a long growth cycle, and its fresh roots contain approximately 75%, making them highly susceptible to mold and deterioration during storage and transportation. This degradation results in significant losses of active compounds, ultimately reducing its medicinal value [5]. Drying, a critical dehydration technique for agricultural products, effectively reduces water activity, inhibits microbial growth, extends shelf life, and preserves the nutritional and bioactive components of material [6,7].

*Licorice* is traditionally dehydrated using sun-drying or shade-drying methods. However, these conventional techniques have limitations such as prolonged drying times (about 3–5 days), severe microbial contamination, and susceptibility to external factors like climatic conditions and environmental pollutants (e.g., dust). Consequently, they fail to meet modern industrial requirements for efficient, stable, and hygienic drying technologies [8]. With advancements in science and technology, modern drying methods, including hot air drying, vacuum drying, microwave drying, and radio frequency drying, have been progressively adopted for *Licorice* processing [9,10]. Each of these technologies has distinct advantages and limitations. While they enhance drying efficiency and help preserve the physicochemical properties of *Licorice* to some extent, challenges such as high energy consumption and significant material shrinkage remain prevalent [11,12]. As intelligent and automated technologies continue to evolve, the drying process for *Licorice* is expected to become more precise and optimized. Concurrently, with deeper exploration of the medicinal properties of *Licorice*, drying technologies will likely focus more on preserving active ingredients and enhancing drying efficiency to better meet market demands. This trend underscores the importance of developing innovative drying techniques that balance efficiency, quality preservation, and environmental sustainability. Studies have demonstrated that ultrasonic pretreatment and RFV heating, as innovative and energy-efficient drying technologies, have demonstrated considerable potential in reducing energy consumption, enhancing the physicochemical properties of agricultural products, and minimizing shrinkage. These technologies offer promising prospects for the industrial-scale development of *Licorice* [13].

Radio frequency (RF) refers to high-frequency alternating current electromagnetic waves with a frequency range of 3 kHz to 300 MHz and a corresponding wavelength range of 1 m to 100 km [14]. A key feature of RF energy is its ability to penetrate material, enabling the simultaneous heating of both its interior and exterior, a phenomenon known as volumetric heating. RF heating methods are generally categorized into two types: induction heating and dielectric heating [15,16]. Compared with traditional drying techniques such as hot air and infrared drying, RFV technology offers several distinct advantages, including non-contact operation, high energy efficiency, and superior product quality [17,18]. Furthermore, the reduced pressure in a vacuum environment significantly lowers the boiling point of water, which facilitates a shorter drying time, improves drying efficiency, minimizes volumetric shrinkage, and effectively inhibits oxidation reactions, preserving the product’s color, flavor, and chemical composition. Currently, radio frequency vacuum (RFV) technology has been extensively utilized in the processing of agricultural products. For instance, Huang et al. [19] investigated changes in drying uniformity and dielectric properties of soybeans during radio frequency heating and developed a mathematical model to describe the transient heating process. Similarly, Guo et al. [20] assessed the impacts of RF drying on the quality and thermodynamic properties of potatoes, comparing it with infrared radiation and microwave drying. Their findings showed that the free radical scavenging ability of potatoes subjected to radio frequency heating was significantly higher than that observed with microwave or infrared drying. Li et al. [21] examined the impacts of novel drying methods (radio frequency and microwave drying) and traditional methods (vacuum, freeze, and hot air drying) on the structural properties and bioactivity of dandelion leaf polysaccharides. The results indicated that materials dried using radio frequency exhibited enhanced antioxidant activity and a pronounced inhibitory effect on α-glucosidase activity in vitro. Additionally, Zhang et al. [22] explored the application of radio frequency drying technology in hickory kernels, analyzing its dielectric properties. Their study revealed that, compared with microwave drying, radio frequency drying significantly increased the drying rate of walnuts, while the dielectric constant and loss factor decreased notably with increasing frequency.

As an efficient and environmentally friendly process, ultrasonic pretreatment technology has attracted significant attention in the drying field in recent years [23]. Ultrasonic waves serve as mechanical energy on the liquid within materials, inducing cavitation effects that accelerate liquid evaporation and enhance drying efficiency. In food drying, ultrasonic pretreatment effectively preserves physical properties while minimizing structural damage to the product [24,25]. With advancements in technology, the potential of ultrasonic pretreatment has been extensively explored across various fields. The continuous optimization of related processes and equipment facilitates the practical application of this technology and lays a foundation for industrial-scale implementation. For example, Pei et al. [26] demonstrated that ultrasonic application improved the quality of far-infrared-dried saffron products by increasing surface microporosity, enhancing capillary formation, reducing mass transfer resistance, and promoting water diffusion in the material. Similarly, Xi et al. [27] found that combining ultrasonic pretreatment with far-infrared drying significantly improved the porous structure of potato slices, enhancing heat and mass transfer during the drying process.

By comparing the effects of different pretreatment parameters, this study aimed to evaluate the impact of ultrasonic pretreatment on the drying characteristics and physicochemical properties of *Licorice*. Furthermore, correlations among various quality indicators were analyzed to identify the optimal dehydration parameters for *Licorice* slices using a combination ultrasonic pretreatment and RFV drying techniques.

## 2. Materials and Methods

### 2.1. Experimental Materials

The *Licorice* used in this study was sourced from a cultivation base in Yuchong County, China. Upon procurement, the *Licorice* roots were wrapped in plastic film and stored in a constant-temperature and -humidity chamber at 5 °C to maintain their integrity. Specimens of uniform size and intact appearance were selected as test materials. The initial moisture content of the samples was determined using a QL-100A moisture analyzer (Xiamen Kewang Electronics Co., Ltd., Xiamen, China) and measured at 53.15% ± 0.3%.

### 2.2. Experimental Equipment and Reagents

A GJS-3-27-JY RF Vacuum Dryer, Hebei Huashi Jiyuan High Frequency Equipment Co., Ltd., Langfang, China; ZL-Z3001 Electronic Balance, Zhonglian Electronic Technology Co., Ltd., Lanzhou, China; KD-0281 Chinese herbal medicine slicer (precision ±0.05 mm), Guangzhou Baiyuan Industrial Co., Ltd., Guangzhou, China; Agilent 1100 High Performance Liquid Chromatograph (HPLC), Agilent Technology Co., Ltd., Santa Clara, CA, USA; CR-410 Colorimeter, Konica Minolta Co., Ltd., Tokyo, Japan; TGL20M High Speed Centrifuge, Hunan Meijiasen Instrument Equipment Co., Ltd., Changsha, China; S-4000N Scanning Electron Microscope, Hitachi Ltd., Tokyo, Japan; T2600S UV Spectrophotometer, Qingdao Jingcheng Instrument Co., Ltd., Qingdao, China.; and THD-T1 ultrasonic cleaning machine, Shenzhen Dianke Equipment Co., Ltd., Shenzhen, China. were used.

Liquiritin apioside, liquiritin, isoliquiritin apioside, isoliquiritin, neoisoliquiritin, liquiritigenin, and licorice chalcone were obtained as standard compounds. The Folin–Ciocalteu reagent, phenol, concentrated sulfuric acid, ascorbic acid, catechin, and acetonitrile were all of analytical grade. All the aforementioned reagents were purchased from Chengdu Pufei De Biotech Co., Ltd. Chengdu, China.

### 2.3. Experimental Methodology

**Figure 1:**
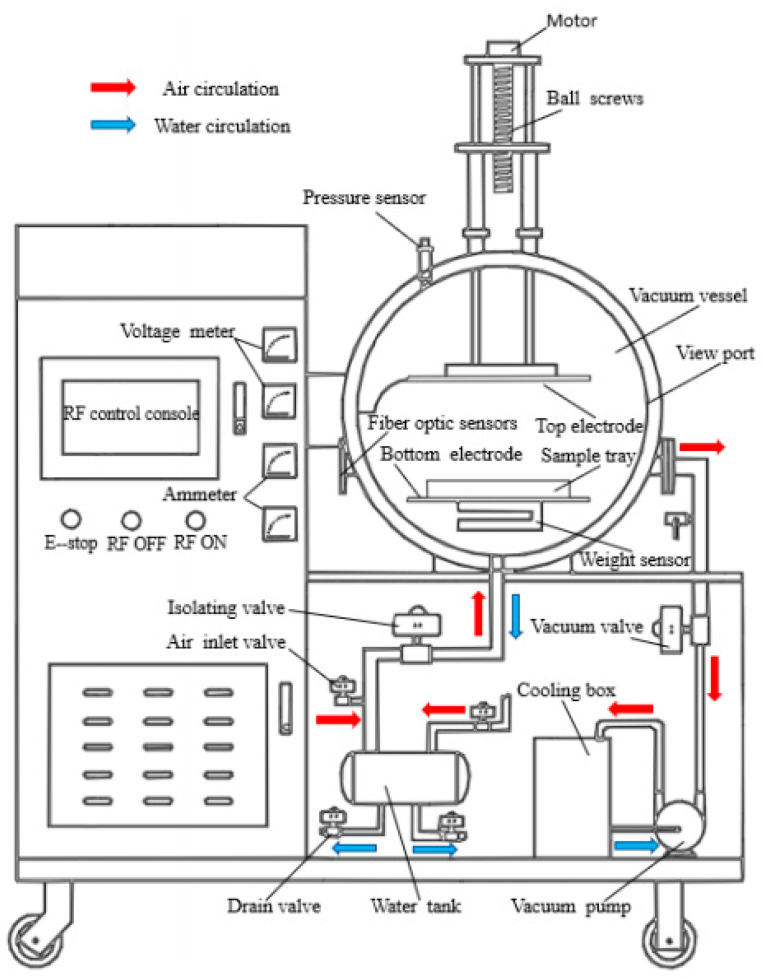
RFV drying equipment illustration.

The experiment was conducted using *Licorice* rhizomes of uniform size and intact shape. The rhizomes were first rinsed to remove surface impurities, trimmed to eliminate excess branches, and then placed in an ultrasonic cleaner containing distilled water for pretreatment under varying conditions, including processing times (20, 30, and 40 min), ultrasonic power levels (120 W, 180 W, and 240 W), and frequencies (20 kHz, 30 kHz, and 40 kHz). Following pretreatment, excess surface water was promptly removed using absorbent paper and the rhizomes were uniformly sliced. A sample weighing 300 ± 0.5 g was spread evenly on a polypropylene porous drying tray and subsequently placed in the radio frequency vacuum (RFV) desiccator for drying (Figure 1). (Note: The *Licorice* slices used in all experiments were consistently maintained at a thickness of 4 mm, with a bed density of 10 g/cm^2^ and an ultrasonic pretreatment water-to-rhizome ratio of 5:1 (*v*/*w*)).

During the drying process, samples were removed at 20 min intervals, weighed, and immediately returned to the desiccator to continue drying. Based on preliminary experiments, the RFV drying conditions were set at a drying temperature of 50 °C, a pole-plate spacing of 90 mm, and a vacuum level of 0.025 MPa. Drying was terminated when the moisture content of the samples dropped below the safe threshold of 15%. The number of experimental replicates and their average values were recorded as the final results. The experimental conditions are summarized in Table 1.

### 2.4. Calculation of Drying Parameters

#### 2.4.1. Dry Basis Moisture Content

The formula for calculating the moisture content of the dry base is as follows [28]:

(1) $$M_{t}=\frac{M_{a}-M_{0}}{M_{0}}\times 100\%$$

where *M*_t_ is the dry basis moisture content, g/g; *M*_a_ is the mass of the *Licorice* slices at moment t, g; and *M*_0_ is the mass of the dry matter of the *Licorice* slices, g.

#### 2.4.2. Moisture Ratio

The formula for calculating the moisture ratio of *Licorice* slices under different drying conditions is as follows [28]:

(2) $$MR=\frac{M_{t}-M_{e}}{M_{0}-M_{e}}$$

where *MR* is the moisture ratio of the *Licorice* slices; *M*_t_ is the dry basis moisture content of the *Licorice* slices at moment t, g/g; and *M*_0_ is the initial dry basis moisture content of the *Licorice* slices, g/g.

#### 2.4.3. Drying Rate

The drying rate of *Licorice* slices was calculated as follows [29]:

(3) $$DR=\frac{M_{t1}-M_{t2}}{t_{2}-t_{1}}$$

where *DR* is the drying rate of the *Licorice* slices; t_1_ and t_2_ are arbitrary drying times; and *M*_t1_ and *M*_t2_ are the dry basis moisture content of the *Licorice* slices at the time of t_1_ and t_2_, respectively, g/g.

#### 2.4.4. Weibull Model

The Weibull distribution function is described by the following expression [7]:

(4) $$MR=exp\left[-({\frac{t}{\alpha })}^{\beta }\right]$$

where α and β are the empirical constants associated with the drying medium; α represents the scale parameter, β represents the shape parameter; and t is the drying time, min.

The fitness of this mathematical model was evaluated using the coefficient of determination R^2^ and the deviation squared χ^2^ and. R^2^ and χ^2^ are expressed as follows [30]:

(5) $$R^{2}=1-\frac{\sum_{i=1}^{N}{\left({MR}_{exp,i}-{MR}_{pre,i}\right)}^{2}}{\sum_{i=1}^{N}({{\bar{MR}}_{exp,i}-{MR}_{pre,i})}^{2}}$$

(6) $$\chi^{2}=\frac{\sum_{i=1}^{N}{\left({MR}_{exp,i}-{MR}_{pre,i}\right)}^{2}}{N-n}$$

where MR_exp,i_ is the Moisture rate measured in the ith test; MR_pre,i_ is the ith predicted moisture rate; and N is the number of groups of experimental data.

#### 2.4.5. Calculation of Effective Moisture Diffusion Coefficient

The transfer characteristics of water in a material can be expressed in terms of the effective water diffusion coefficient. The commonly used formula for calculating the effective water diffusion coefficient from Fick’s second law of diffusion is as follows [7]:

(7) $$\ln MR=\ln\frac{8}{\pi^{2}}-\frac{\pi^{2}D_{eff}}{L^{2}}t$$

where *D_eff_* is the effective water diffusion coefficient of the material; *L* is the thickness of the material layer of the *Licorice* slices, mm; and t is the drying time, s. The *Licorice* slices used in the experiment were cylindrical with a thickness of 4 mm.

(8) $$D_{cal}=\frac{L^{2}}{\alpha }$$

where *D_cal_* is estimated water diffusion coefficient; the relationship between D*_cal_* and *D_eff_* is as follows [12]:

(9) $$D_{eff}=\frac{D_{cal}}{R_{g}}$$

where *R_g_* is the parameter related to geometry.

### 2.5. Measurement of Quality Indicators

#### 2.5.1. Determination of Color

The surface color of fresh *Licorice* slices and samples subjected to different drying conditions was measured using a colorimeter [18]. The color difference between the tested samples was expressed as ∆*E*, where a smaller ∆*E* value indicates higher quality of the dried products. ∆*E* was calculated according to the following formula [30,31]:

(10) $$∆E=\sqrt{{\left(L^{*}-L_{0}\right)}^{2}+{\left(a^{*}-a_{0}\right)}^{2}+{\left(b^{*}-b_{0}\right)}^{2}}$$

(11) $$H^{0}={tan}^{-1}\left(\frac{b^{*}}{a^{*}}\right)$$

(12) $$Chroma=\sqrt{{a^{*}}^{2}+{b^{*}}^{2}}$$

where L*, a*, and b* are the brightness, red-green value, and blue-yellow value of the dried *Licorice* products; *L*_0_, *a*_0_, and *b*_0_ are the brightness, red/green value, and blue/yellow value of fresh samples.

#### 2.5.2. Total Phenolic, Polysaccharide and Total Flavonoid Extract Preparation

*Licorice* slices were ground into powder and sieved through an 80-mesh sieve. Precisely 1.0 g of the sample powder was weighed using an analytical balance and placed into a conical flask containing 25 mL of 75% anhydrous ethanol. The mixture was subjected to rotary shaking at 120 r·min^−1^ for 48 h under light-protected, room temperature conditions. Following the extraction, the sample was centrifuged at 5000 r·min^−1^ for 10 min, and the supernatant was collected. The volume of the extract was adjusted to 25 mL with anhydrous ethanol, and the solution was stored at 4 °C for subsequent analysis of total phenolics, antioxidant activity, soluble sugars, and total flavonoids. To minimize experimental error, each test was performed in triplicate and the average value was recorded as the final result.

#### 2.5.3. Determination of Total Phenolic Content

The total phenol content was determined using the Folin–Ciocalteu method. The total phenol content was standardized using gallic acid as the control standard. The total phenol content was calculated using the following formula [32]:

(13) $$TPC=(V_{T}\times C_{2})/(V_{M}\times M)$$

where *TPC* is the total phenolic content, mg/g; *V_T_* is the total volume of the sample extract, mL; *C*_2_ is sucrose mass concentration; *V_M_* is the volume of sample extract used in the titration, mL; and *M* is the mass of dry matter of sample, g.

#### 2.5.4. Determination of Polysaccharide Content

The polysaccharide content of *Licorice* slices was determined by the sulfuric acid–phenol method. Sucrose was used as a reference substance and no sample solution was used as a blank control.

(14) $$P_{c}=(V_{T}\times C_{1})/(V_{M}\times M)$$

where *P_c_* is the soluble sugar mass concentration, g/g.

#### 2.5.5. Determination of Total Flavonoids Content

The determination of total flavonoids was performed using NaNO_2_-Al(NO_2_)_3_-NaOH [31]. The total flavonoid content was standardized using catechin as a control standard. The total flavonoid content was calculated as follows:

(15) $$TFC=(V_{T}\times C_{3})/(V_{M}\times M)$$

where *TFC* is the mass concentration of catechin, g/g.

#### 2.5.6. Microstructure

Before performing scanning electron microscopy (SEM), the samples were cut into 5 mm × 5 mm sections and then immediately fixed with 2.5% glutaraldehyde solution to stabilize the structure and composition of the biological system. The samples after gold spraying were observed by SEM with an accelerating voltage of 5.0 kV.

#### 2.5.7. Determination of Natural Active Substances

Preparation of test samples: Each powdered sample was passed through an 80-mesh sieve and 0.5 g of the sieved powder was accurately weighed. The sample was transferred into a stoppered conical flask containing 25 mL of 80% anhydrous ethanol and subjected to ultrasonic treatment (100 W, 40 kHz) for 25 min. After treatment, the solution volume was adjusted to 25 mL with anhydrous ethanol, centrifuged at 5000 r·min^−1^ for 10 min, and the supernatant was filtered through a 0.22 μm membrane filter for subsequent analysis.

Chromatographic conditions: The analysis was performed on an Agilent-C18 chromatographic column (250 mm × 4.6 mm, 5 μm). The mobile phase consisted of acetonitrile (B) and 1% acetic acid aqueous solution (D). The gradient elution program was as follows: 0–4 min, 15–40% B; 4–8 min, 4–65% B; 8–10 min, 65–85% B; 10–12 min, 85–15% B; and 12–16 min, 15% B. The mobile phase flow rate was maintained at 1.0 mL·min^−1^, the column temperature was set at 40 °C, and the detection wavelength was 250 nm. A sample injection volume of 1 μL was used, with a sample loading volume of 2 μL.

### 2.6. Statistical Analysis

Each group of experiments was repeated three times and the average value was taken as the final result. The calculation of the moisture ratio and drying rate was done by Excel software, while the plotting of graphs and bar graphs were processed by Origin 8.0. The analysis of variance (ANOVA) of the data was performed by SPSS 24.0 software and the significance test of the difference in means was performed by Tukey’s multiple-range method with the significance level set at 0.05.

## 3. Results and Discussion

### 3.1. Effect of Different Ultrasonic Pretreatment Conditions on Drying Characteristics of Licorice Slices

#### 3.1.1. Effect of Ultrasonic Pretreatment Time on Drying Characteristics of Licorice Slices

**Figure 2:**
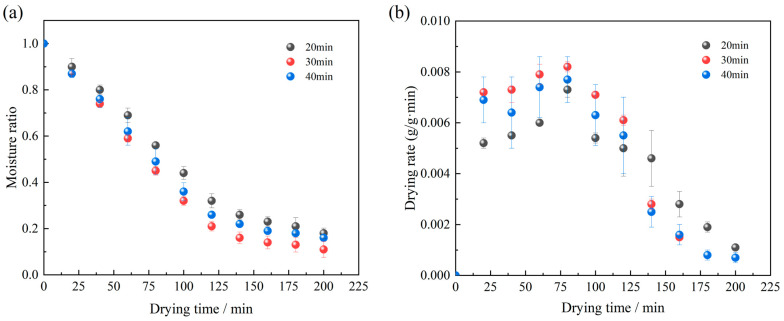
Effect of ultrasonic pretreatment time on drying curves (**a**) and drying rate curves (**b**) of *Licorice* slices.

The effect of different ultrasonic pretreatment durations on the radio frequency vacuum (RFV) drying characteristics of *Licorice* slices, at an ultrasonic frequency of 40 kHz and a power of 180 W, is illustrated in Figure 2.

The results indicate that the drying time required to achieve a safe moisture content decreased significantly, from 200 min to 130 min. Among the tested durations, ultrasonication for 20 min resulted in the lowest drying rate and the longest drying time (200 min) compared to 30 and 40 min. The drying rates under ultrasonic pretreatment durations of 20, 30, and 40 min were 1.04 g/g·min, 1.27 g/g·min, and 1.14 g/g·min, respectively, representing increases of 14.29%, 39.56%, and 25.27% compared to RFV drying alone. This phenomenon may be attributed to the relationship between sonication duration and water distribution within the *Licorice* slices. Longer ultrasonic treatment may facilitate the infiltration of water into the interstitial spaces and cellular interiors. Water in the interstitial spaces is readily removed during drying, whereas intracellular water presents greater resistance to evaporation, as it must first diffuse through the cell membrane to reach the sample surface. Consequently, an optimal ultrasonic pretreatment duration can significantly enhance drying efficiency by balancing water redistribution and removal processes [8].

#### 3.1.2. Effect of Ultrasonic Power on Drying Characteristics of Licorice Slices

**Figure 3:**
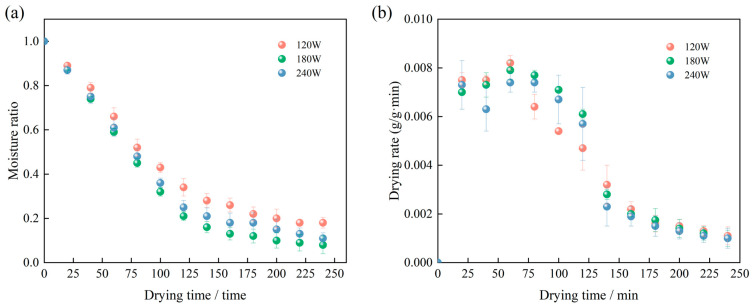
Effect of ultrasonic power on drying curves (**a**) and drying rate curves (**b**) of *Licorice* slices.

The effects of varying ultrasonic pretreatment power on the RFV drying characteristics of *Licorice* slices at an ultrasonic frequency of 40 kHz and a pretreatment duration of 30 min are presented in Figure 3. The RFV drying conditions were maintained at a vacuum pressure of 0.025 MPa, a drying temperature of 60 °C, and an electrode plate spacing of 90 mm. Under these conditions, the influence of ultrasonic power on the moisture content and drying rate of *Licorice* slices was evaluated. As shown in Figure 3, increasing the ultrasonic power resulted in a shorter drying time to achieve a moisture content below the safe threshold. At an ultrasonic power of 180 W, the moisture ratio decreased more rapidly compared to 120 W. This enhancement was attributed to the higher ultrasonic energy at greater power levels, which generates micro-jets and increases the medium’s temperature. These effects enhance cavitation and mechanical actions, facilitating water migration and accelerating its removal from the material. Thus, higher ultrasonic power contributes to improved drying efficiency through intensified water loss mechanisms.

#### 3.1.3. Effect of Ultrasonic Frequency on Drying Characteristics of Licorice Slices

**Figure 4:**
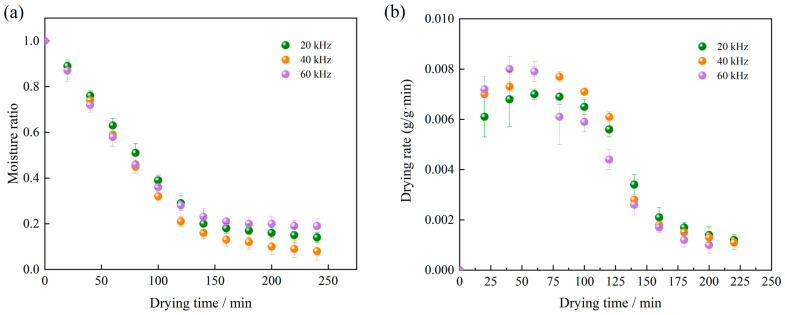
Effect of ultrasonic frequency on drying curves (**a**) and drying rate curves (**b**) of *Licorice* slices.

The effects of different ultrasonic pretreatment frequencies on the RFV drying characteristics of *Licorice* slices, under a vacuum pressure of 0.025 MPa, a drying temperature of 60 °C, and an electrode plate spacing of 90 mm, are presented in Figure 4. As shown in Figure 4, increasing the ultrasonic frequency influenced the moisture content and drying rate of the *Licorice* slices during RFV drying. The shortest time required to achieve a safe moisture content was observed at 40 kHz, which also exhibited a relatively high drying rate. This phenomenon can be attributed to the cavitation effect induced by ultrasonication, which generates rapid and alternating cycles of compression and expansion. These cycles reduce surface tension on the material and promote the formation of microchannels within the structure of the *Licorice* slices. These microchannels enhance water diffusion pathways, facilitating increased surface evaporation rates. These findings provide a critical foundation for optimizing ultrasonic pretreatment parameters, enabling improvements in drying efficiency and reductions in overall dehydration time.

### 3.2. Effect of Ultrasonic Pretreatment on Effective Water Diffusion Rate of Licorice Slices

To comprehensively investigate the heat and mass transfer properties of water within the material, the effective water diffusion coefficient of *Licorice* slices during the drying process was determined experimentally. This parameter is critical for elucidating the water migration mechanism during drying and for optimizing the drying process. The calculated parameters for the far-infrared drying of *Licorice* slices under various conditions are summarized in Table 2. The estimated water diffusion coefficients (*D*_cal_) ranged from 1.018 × 10^−7^ to 1.865 × 10^−7^, while the effective water diffusion coefficients were in the range of 1.972 × 10^−10^ to 3.729 × 10^−10^. These values generally increased with decreasing drying temperature, pole-plate spacing, and vacuum pressure. This trend can be attributed to the fact that RFV drying was primarily governed by the diffusion rate of water within the material. Higher drying temperatures, lower vacuum pressures, and reduced pole-plate spacing enhance the transfer of water from the material’s solid phase to its surface, thereby increasing the water diffusion coefficient.

### 3.3. Effect of Ultrasonic Pretreatment on the Color of Licorice Slices

Color is a critical parameter for assessing the drying quality of materials. Table 3 presents the impact of RFV drying on the color attributes of Licorice under various pretreatment conditions. Results showed that the a* values of the pre-treated RFV-dried *Licorice* were all lower than that of the natural drying condition (2.18), and its skin brightness (L*) was close to that of the natural drying condition. Since the total color difference (Δ*E*) of natural drying was smaller than that of the pretreated RFV drying condition, this suggested that the pretreatment may have affected the browning response of RFV-dried *Licorice* to some extent. The brightness (L*) and red-green value (a*) of the pretreated *Licorice* were lower than those of the naturally dried samples. According to the data in Table 3, as the ultrasonic treatment frequency increased from 20 kHz to 60 kHz, the Δ*E* value decreased from 12.35 to 11.06, indicating that the increase in ultrasonic frequency helped to inhibit the browning response of *Licorice*. In addition, the Δ*E* values showed a tendency of decreasing and then increasing with the increase of ultrasonic treatment time and ultrasonic power, which indicated that the color of dried *Licorice* product was better at the ultrasonic power of 180 W and ultrasonic frequency of 40 kHz. Prolonged ultrasonication may result in an increased gap between cells and the formation of additional micropores, which, while enhancing the effective contact area with the ultrasonic medium, simultaneously damages the material’s surface and intensifies color changes. A comprehensive analysis of the effect of pretreatment conditions on the color of *Licorice* found that the Δ*E* value of *Licorice* was lower and the color was good at the ultrasonication time of 30 min, power of 180 W, and frequency of 40 kHz.

### 3.4. Effect of Ultrasonic Pretreatment on Total Phenol Content of Licorice Slices

**Figure 5:**
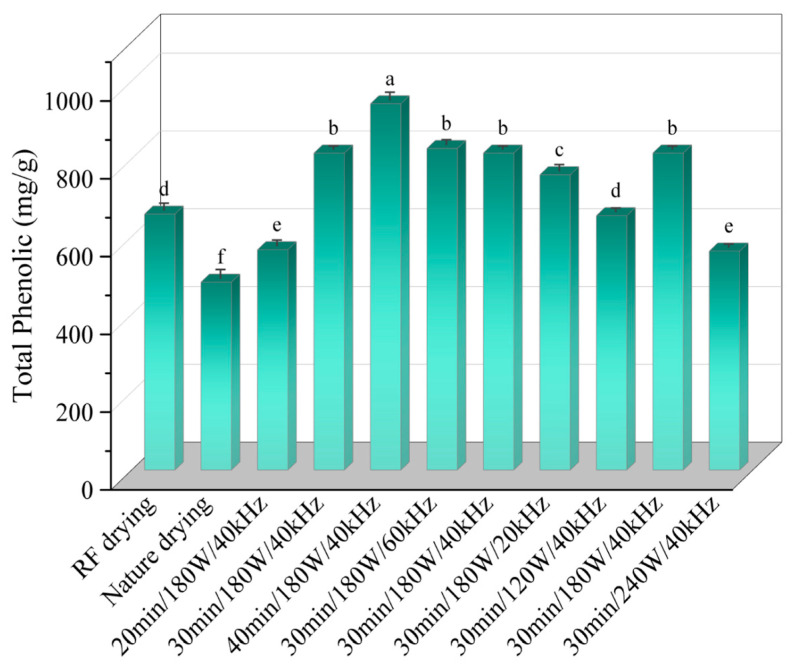
Effects of different drying conditions on total phenolics of *Licorice* slices. Note: A different lowercase letter after each column indicates a significant difference (*p* < 0.05).

The effects of various ultrasonic pretreatment conditions on the total phenol content in *Licorice* slices are presented in Figure 5. The total phenol content under different ultrasonic pretreatment conditions ranged from 483.33 ± 3.48 to 941.94 ± 4.06 mg/100 g, with the observed variation pattern illustrated in Figure 5. As indicated in the figure, an increase in ultrasonic pretreatment time was associated with a gradual rise in total phenol content, which reached values of 566.64 mg/100 g, 815.23 mg/100 g, and 941.94 mg/100 g at 20, 30, and 40 min of treatment, respectively. Conversely, the total phenol content showed a decreasing trend with increasing ultrasonic power. At ultrasonic powers of 120 W, 180 W, and 240 W, the total phenol content was 826.94 mg/100 g, 815.23 mg/100 g, and 759.45 mg/100 g, respectively, representing increases of 20%, 19%, and 13% compared to the control (658.23 mg/100 g) without ultrasound treatment. Zang et al. [32] reported that increased ultrasonic power enhances the polysaccharide content in *Licorice* slices, with soluble sugars promoting polyphenol accumulation and maintaining the balance of flavanol polyphenolic acid. Similar findings were observed in this study, suggesting that ultrasound treatment supports the retention of total phenolic content in *Licorice*. This could be attributed to the mechanical and cavitation effects of ultrasound, which cleave covalently bound polyphenolic compounds, facilitating their release from the cell wall and thereby increasing the total phenolic content [33]. In terms of ultrasonic frequency, the total phenolic content initially increased and then decreased with rising frequency, with values of 654.10 mg/100 g, 815.23 mg/100 g, and 563.20 mg/100 g observed at 20 kHz, 40 kHz, and 60 kHz, respectively. The decrease in phenolic content at higher frequencies may be due to mechanical damage to the internal cell walls caused by high-intensity ultrasound leading to the leakage of oxidase and peroxidase with greater activity. Furthermore, during RFV drying, strong pressure, shear forces, and temperature and humidity gradients within the material may induce oxidation of heat-sensitive phenolic compounds, accelerating their degradation. Therefore, the total phenolic content in *Licorice* slices was best preserved under ultrasonic pretreatment conditions of 30 min, 180 W, and 40 kHz.

### 3.5. Effect of Ultrasonic Pretreatment on Polysaccharide Content of Licorice Slices

**Figure 6:**
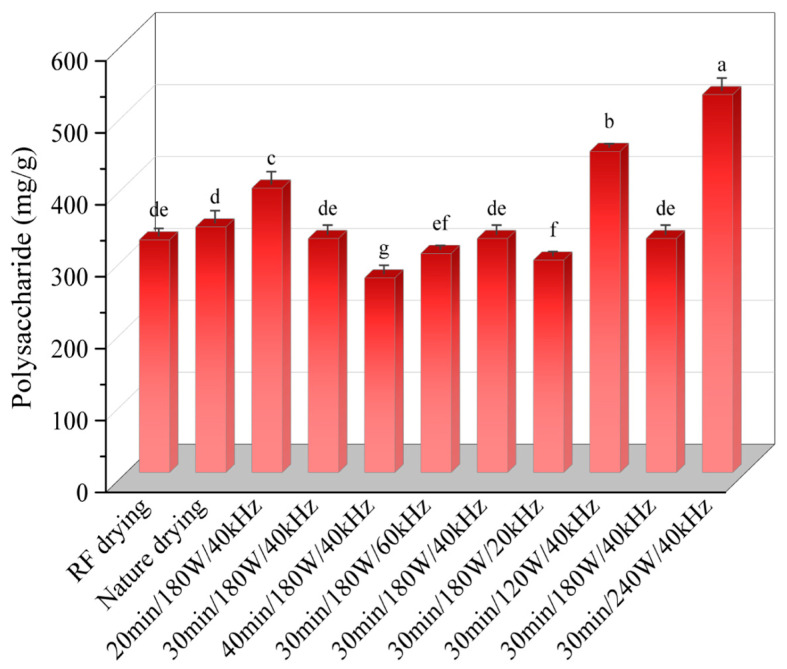
Effects of different drying conditions on polysaccharides of *Licorice* slices. Note: A different lowercase letter after each column indicates a significant difference (*p* < 0.05).

The effects of various ultrasonic pretreatment conditions on the polysaccharide content in *Licorice* slices are presented in Figure 6. As shown in the figure, ultrasonic pretreatment significantly influenced the polysaccharide content (*p* < 0.05), with a notable increase in polysaccharide levels following treatment. The polysaccharide content exhibited a trend of initially increasing and then decreasing with the extension of ultrasonic pretreatment time. Specifically, the content was 395.51 mg/100 g, 325.36 mg/100 g, and 270.58 mg/100 g after 20, 30, and 40 min of pretreatment, respectively. This trend may be attributed to the thermal effect of ultrasonic waves, which raised the temperature of the medium during the pretreatment process. As a result, glycosides, including polysaccharides, are more likely to undergo Maillard reactions with proteins at elevated temperatures, leading to the degradation of polysaccharide content in the *Licorice* slices. Regarding ultrasonic power, the polysaccharide content followed a similar increasing-then-decreasing trend. This can be explained by the fact that increasing ultrasonic power enhanced the mechanical and cavitation effects, reducing mass transfer resistance within the material, promoting water diffusion, shortening drying time, and improving drying rates. Consequently, this reduced the duration of the Maillard reaction. However, as ultrasonic power increased further, the increase in polysaccharide content diminished and eventually reversed, possibly due to the intensified thermal effects of ultrasound, which rapidly raised the local temperature, promoting the thermal degradation of saccharides and leading to greater oxidative reactions and a reduction in polysaccharide content. As ultrasonic frequency increased, the polysaccharide content initially increased but then declined, with values of 446.51 mg/100 g, 325.36 mg/100 g, and 525.56 mg/100 g at different frequencies, respectively. This negative trend at higher frequencies could be attributed to the stronger pressure, stress, and shear forces generated by ultrasonic waves, which disrupt the polysaccharide structure, including the cleavage of side chains, leading to a decrease in polysaccharide content. In conclusion, ultrasonic pretreatment significantly improved the polysaccharide content in *Licorice* slices compared to RFV drying alone (35.07 mg/100 g), indicating that ultrasonic pretreatment positively impacts polysaccharide retention. This effect may be due to the disruption of carbohydrate metabolic equilibrium within the *Licorice* slices caused by the cavitation and mechanical effects of ultrasound. However, the underlying mechanisms require further investigation.

### 3.6. Effect of Ultrasonic Pretreatment on Total Flavonoid Content of Licorice Slices

**Figure 7:**
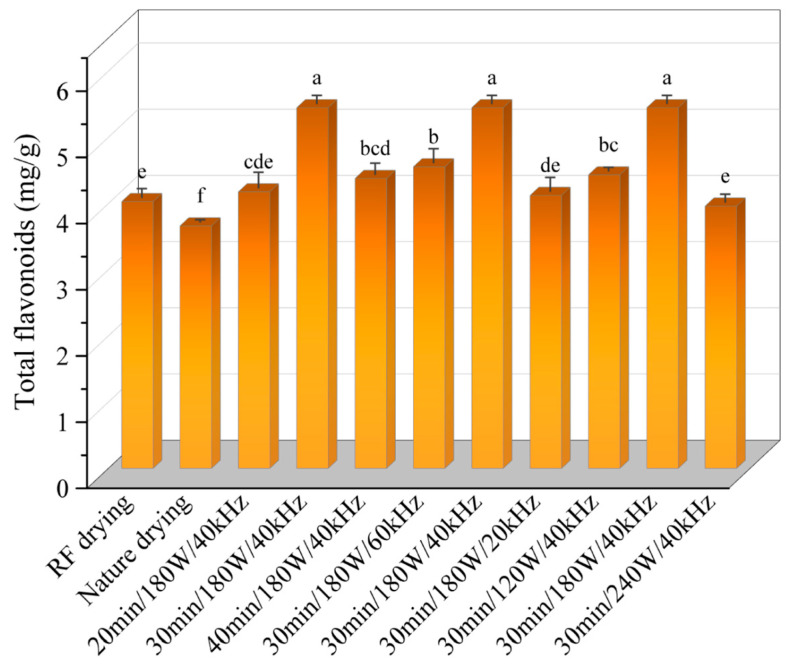
Effects of different drying conditions on total flavonoids of *Licorice* slices. Note: A different lowercase letter after each column indicates a significant difference (*p* < 0.05).

The effects of various ultrasonic pretreatment conditions on the total flavonoid content are illustrated in Figure 7. As ultrasonic pretreatment time and power increased, the total flavonoid content initially increased before subsequently decreasing. At an ultrasonic pretreatment time of 30 min, the total flavonoid content reached 5.45 mg/100 g, representing a 26.03% increase compared to the RFV drying process alone. When comparing different pretreatment durations, a 40 min ultrasonic pretreatment resulted in a 19.6% reduction in flavonoid content, indicating that prolonged ultrasonic treatment is detrimental to flavonoid retention. This decline may be attributed to the thermal effects induced by ultrasound, which could disrupt the structural integrity and biological activity of flavonoids, accelerating their degradation [34]. Ultrasonic power also significantly influenced the total flavonoid content. The content values were 4.43 mg/100 g, 5.45 mg/100 g, and 3.96 mg/100 g at ultrasonic power levels of 120 W, 180 W, and 240 W, respectively. With increasing ultrasonic power, the enhanced thermal and kinetic energy within the material likely exacerbated the degradation of heat-sensitive phenolic compounds and flavonoids, leading to a reduction in total flavonoid content. The effect of ultrasonic frequency on the total flavonoid content exhibited a trend similar to that of power. The highest flavonoid content of 5.45 mg/100 g was observed at an ultrasonic frequency of 40 kHz, which was 19.6% and 32.1% higher than that at 20 kHz and 60 kHz, respectively. This suggests that a moderate increase in ultrasonic frequency may contribute to improved flavonoid retention. Optimal selection of ultrasonic pretreatment time, power, and frequency can effectively enhance flavonoid retention. However, excessively long treatment times or high power levels may have an adverse effect on flavonoid preservation.

### 3.7. Microstructure Analysis

**Figure 8:**
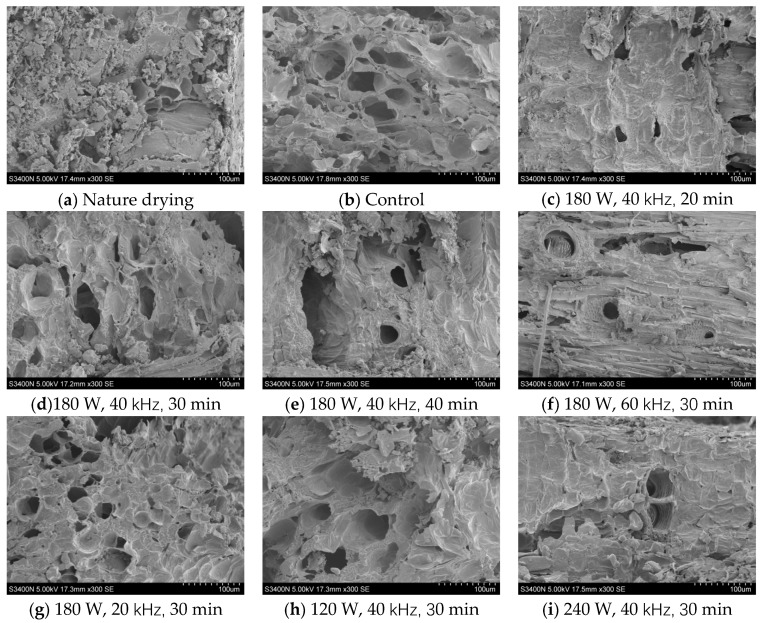
Effects of different drying conditions on the microstructure of *Licorice* slices.

The microstructures of dried products under different ultrasonic pretreatment conditions are shown in Figure 8. It was found that the internal tissue structure of *Licorice* slices continue to shrink and fragment during the drying process and the internal microporous channel structure was destroyed, resulting in smaller cavities. Among them, the cellular tissue structure of naturally dried products (Figure 8a) and products dried without ultrasound pretreatment (Figure 8b) was more compact and dense, and the surface of the naturally dried samples was more fragmented and raised, with a relatively smaller number of internal pores. An ultrasonic frequency of 40 kHz and ultrasonic time of 30 min revealed that the higher the ultrasonic power, the clearer and more neatly arranged microchannels on the surface of *Licorice* slices (Figure 8c–e). At an ultrasonic power of 180 W and time of 30 min, the microchannels of the cellular structure on the surface of the samples changed less with changes in frequency, indicating that the ultrasonic frequency did not have a significant effect on their microstructure. In addition, with the increase of ultrasonication time, the deformation of the honeycomb structure was severe, the cells appeared to be broken and collapsed, and the prolonged ultrasonication reduced the integrity of the sample tissue cells (Figure 8d,h,i). At the ultrasonic power of 240 W, ultrasonic frequency of 40 kHz, and ultrasonic time of 30 min, the microstructure of the sample microstructure was loosened and the number of closed or semi-closed microporous channels was reduced, the adjacent cell walls were complete and permeable and arranged in an orderly manner, a large number of cellular pore structures appeared, and the pore shrinkage and fragmentation were low. At this time, the skeleton structure of the cells was kept in good condition and the microchannels were visible, and the fiber reticulation structure could be observed under this condition. Under this condition, the fiber mesh structure could be observed.

### 3.8. Analysis of Active Ingredient Content

The effects of ultrasonic pretreatment radio frequency drying on the content of the effective active components of *Licorice* slices are shown in the Table 4. The degree of influence of different ultrasound conditions on the effective components varied significantly. Under the condition of ultrasonic time of 30 min, compared to the control group, neoisoliquiritin, isoliquiritin, and liquiritigenin decreased by 9.88%, 3.48%, and 2.95%, respectively, and apigenin and liquiritigenin increased by 32.44% and 37.95%, respectively, while the contents of other active ingredients did not change significantly, when the ultrasonic power was increased from 120 W to 240 W. The contents of liquiritigenin, *Licorice* chalcone, isoliquiritin apioside, and liquiritin showed an increasing trend, while the others showed an increasing and then decreasing trend.

## 4. Discussion

Ultrasonic pretreatment significantly enhanced the drying performance and physicochemical properties of *Licorice* slices during the RFV drying process. Compared to RFV drying alone, ultrasonic pretreatment reduced the drying time by 12.39% to 31.89%, thereby markedly improving drying efficiency. The drying rate increased notably with longer ultrasonic treatment durations, higher power levels, and greater frequencies. This improvement can primarily be attributed to the cavitation effect and mechanical vibrations induced by ultrasound. The collapse of cavitation bubbles enhanced cell wall permeability and created additional pathways for water migration, while mechanical vibrations weakened the binding strength between water molecules and the material matrix, facilitating the diffusion of internal moisture to the surface.

Furthermore, ultrasonic pretreatment significantly increased the contents of liquiritin apioside, isoliquiritin apioside, neoisoliquiritin, polysaccharides, total phenolics, and total flavonoids in *Licorice* slices, indicating its positive role in protecting and releasing functional compounds. The treatment also improved the color quality of the samples and optimized their microstructure by enhancing internal porosity and preventing cellular collapse, resulting in a more uniform structure after drying. However, excessive ultrasonic treatment parameters (such as prolonged treatment durations, high frequencies, or high power levels) led to structural damage, including excessive cell rupture, which negatively affected the physicochemical properties of the samples. These adverse effects are likely due to excessive mechanical stress or localized overheating caused by intense cavitation, necessitating careful parameter control in practical applications.

Based on experimental results, the optimal ultrasonic pretreatment parameters were determined to be a treatment duration of 30 min, a power level of 180 W, and a frequency of 40 kHz. These conditions effectively balanced an improved drying performance with the preservation of the physicochemical quality of *Licorice* slices.

This study demonstrates that ultrasonic pretreatment, when combined with RFVD, represents an efficient and energy-saving drying technology. It enhances drying efficiency while maintaining or even improving the functional compound retention and sensory quality of the final product. This approach offers valuable technical support for the deep processing of medicinal plants such as *Licorice* and introduces new strategies for optimizing drying processes in the food and pharmaceutical industries. Moreover, its advantages in energy consumption, product quality, and environmental sustainability contribute significantly to advancing the modernization of traditional drying technologies.

## Figures and Tables

**Table 1:** Experimental conditions of ultrasonically pretreated RFV-dried *Licorice* slices.

Experiment Number	Experimental Factors		
	Ultrasonic Time/Min	Ultrasonic Power/W	Ultrasonic Frequency/kHz
1	20	180	40
2	30	180	40
3	40	180	40
4	30	120	40
5	30	240	40
6	30	180	20
7	30	180	60

**Table 2:** Effective water diffusion rate of *Licorice* slices subjected to various drying conditions.

Experiment Number	D_eff_ (10^−10^ m^2^ s^−1^)	D_cal_ (10^−7^ m^2^ s^−1^)	R_g_	α	β	R^2^	χ^2^ (×10^−5^)
20 min-180 W-40 kHz	1.972	1.018	528.992	155.478	1.178	0.9997	2.381
30 mim-180 W-40 kHz	2.648	1.374	518.858	116.460	1.182	0.9992	7.373
40 min-180 W-40 kHz	3.729	1.865	500.167	85.794	1.268	0.9991	9.730
30 min-180 W-60 kHz	1.972	1.018	516.340	157.106	1.220	0.9984	10.326
30 mim-180 W-40 kHz	2.648	1.374	518.858	116.424	1.181	0.9992	7.373
30 min-180 W-20 kHz	2.756	1.425	517.092	112.275	1.212	0.9992	9.240
30 min-120 W-40 kHz	2.128	1.078	506.542	83.504	1.218	0.9991	9.976
30 mim-180 W-40 kHz	2.648	1.374	518.858	116.460	1.182	0.9992	7.373
30 min-240 W-40 kHz	3.335	1.742	522.399	143.490	1.196	0.9991	7.536

**Table 3:** Color of *Licorice* slices subjected to various drying conditions.

Drying Condition	L*	a*	b*	∆*E*	*H* ^0^	C
Nature drying	89.56 ± 0.03	2.18 ± 0.06	12.71 ± 0.05	12.25 ± 0.03	1.40	12.90
RFV	84.45 ± 0.12	1.38 ± 0.07	11.93 ± 0.15	10.48 ± 0.17	1.46	12.01
20 min/180 W/40 kHz	89.74 ± 0.10	1.94 ± 0.04	13.12 ± 0.08	12.02 ± 0.10	1.42	13.26
30 mim/180 W/40 kHz	88.68 ± 0.45	1.67 ± 0.12	13.00 ± 0.42	11.42 ± 0.61	1.44	13.11
40 min/180 W/40 kHz	89.36 ± 0.03	2.00 ± 0.03	13.45 ± 0.06	11.54 ± 0.03	1.42	13.60
30 min/180 W/20 kHz	88.73 ± 0.12	1.76 ± 0.02	11.89 ± 0.09	12.35 ± 0.10	1.42	12.02
30 mim/180 W/40 kHz	88.68 ± 0.45	1.67 ± 0.12	13.00 ± 0.42	11.42 ± 0.61	1.44	13.11
30 min/180 W/60 kHz	89.36 ± 0.10	1.93 ± 0.04	14.09 ± 0.08	11.06 ± 0.04	1.43	14.22
30 min/120 W/40 kHz	89.07 ± 0.12	1.93 ± 0.03	13.31 ± 0.19	11.44 ± 0.21	1.43	13.45
30 mim/180 W/40 kHz	88.68 ± 0.45	1.67 ± 0.12	13.00 ± 0.42	11.42 ± 0.61	1.44	13.11
30 min/240 W/40 kHz	88.58 ± 0.26	2.07 ± 0.08	12.55 ± 0.15	11.75 ± 0.28	1.41	12.72

**Table 4:** Natural active substances content of *Licorice* slices subjected to various drying conditions.

Drying Condition	Liquiritin Apioside	Liquiritin	Isoliquiritin Apioside	Isoliquiritin	Neoisoliquiritin	Liquiritigenin	Licorice Chalcone
RFV	2.497 ± 0.008 ^c^	1.159 ± 0.017 ^ab^	5.007 ± 0.005 ^ab^	5.052 ± 0.006 ^b^	6.093 ± 0.008 ^a^	6.157 ± 0.009 ^a^	5.167 ± 0.009 ^d^
Nature drying	2.506 ± 0.179 ^c^	0.235 ± 0.169 ^b^	4.877 ± 0.031 ^ab^	5.116 ± 0.024 ^ab^	5.609 ± 0.010 ^bc^	5.993 ± 0.005 ^ab^	5.475 ± 0.017 ^a^
20 min/180 W/40 kHz	3.445 ± 0.007 ^a^	2.483 ± 1.633 ^a^	5.096 ± 0.054 ^a^	4.934 ± 0.080 ^b^	5.537 ± 0.093 ^bcd^	5.748 ± 0.015 ^cd^	5.284 ± 0.028 ^cd^
30 mim/180 W/40 kHz	3.969 ± 0.122 ^abc^	2.268 ± 1.034 ^ab^	5.033 ± 0.028 ^ab^	4.882 ± 0.036 ^b^	5.545 ± 0.043 ^bcd^	5.980 ± 0.066 ^d^	5.346 ± 0.099 ^cd^
40 min/180 W/40 kHz	3.282 ± 0.079 ^ab^	2.099 ± 0.560 ^b^	4.914 ± 0.182 ^b^	5.514 ± 0.057 ^a^	5.651 ± 0.020 ^bcd^	6.005 ± 0.006 ^ab^	5.309 ± 0.014 ^bc^
30 min/180 W/20 kHz	2.980 ± 0.337 ^abc^	2.784 ± 0.236 ^a^	4.901 ± 0.104 ^ab^	5.517 ± 0.038 ^a^	5.475 ± 0.003 ^bcd^	5.429 ± 0.194 ^abc^	5.286 ± 0.047 ^cd^
30 mim/180 W/40 kHz	3.969 ± 0.122 ^abc^	2.268 ± 1.034 ^ab^	5.033 ± 0.028 ^ab^	4.882 ± 0.036 ^b^	5.545 ± 0.043 ^bcd^	5.980 ± 0.066 ^d^	5.346 ± 0.099 ^cd^
30 min/180 W/60 kHz	2.780 ± 0.063 ^bc^	1.076 ± 0.678 ^ab^	4.731 ± 0.226 ^b^	5.153 ± 0.049 ^ab^	5.498 ± 0.147 ^bcd^	5.798 ± 0.037 ^bcd^	5.292 ± 0.040 ^bc^
30 min/120 W/40 kHz	2.937 ± 0.511 ^abc^	2.017 ± 0.842 ^ab^	5.008 ± 0.161 ^ab^	5.060 ± 0.256 ^b^	5.418 ± 0.037 ^d^	5.641 ± 0.190 ^a^	5.259 ± 0.078 ^cd^
30 mim/180 W/40 kHz	3.969 ± 0.122 ^abc^	2.268 ± 1.034 ^ab^	5.033 ± 0.028 ^ab^	4.882 ± 0.036 ^b^	5.545 ± 0.043 ^bcd^	5.980 ± 0.066 ^d^	5.346 ± 0.099 ^cd^
30 min/240 W/40 kHz	3.277 ± 0.418 ^ab^	2.540 ± 0.753 ^a^	5.042 ± 0.147 ^ab^	5.051 ± 0.456 ^b^	5.494 ± 0.072 ^cd^	5.991 ± 0.034 ^ab^	5.410 ± 0.038 ^ab^

Note: A different lowercase letter after each column indicates a significant difference (*p* < 0.05).

## Data Availability

The original contributions presented in this study are included in the article. Further inquiries can be directed to the corresponding author.

## References

[1] Kamali M., Shabanpour B., Pourashouri P., Pourashouri P., Kordjazi M. (2024). Evaluating shelf life and anti-browning of shrimp by chitosan-coated nanoliposome loaded with *Licorice* root extract. Food Chem. X.

[2] Zhu L., Xie Y., Li M., Zhang X., Ji X., Zhang X., Zhu H., Gu J., Zhang Q., Yang X. (2024). Design and optimization of heat pump with infrared drying for *Glycyrrhiza uralensis* (*Licorice*) processing. Front. Nutr..

[3] Goudarzi T., Tabrizi L., Nazeri V., Etemadi M. (2024). Nutrient distribution in various tissues of Licorice (*Glycyrrhiza glabra* L.) and the influence of soil fertility on the levels of its bioactive compounds. Ind. Crops Prod..

[4] Manami S., Yeon C.S., Kentaro F., Mareshige F., Toshiya F., Hikaru S. (2023). Disruption of a Licorice cellulose synthase-derived glycosyltransferase gene demonstrates its in planta role in soyasaponin biosynthesis. Plant Cell Rep..

[5] Hu D., Yang G., Liu X., Qin Y., Zhang F., Sun Z., Wang X. (2024). Comparison of different drying technologies for coffee pulp tea: Changes in color, taste, bioactive and aroma component. LWT-Food Sci. Technol..

[6] Sun W., Li M., Zhang Y., Ai Z., Lei D., Pei Y., Liu Y. (2023). Effect of different drying techniques on drying characteristics, physical quality, and active components of Citri reticulatae pericarpium, and the correlation between physiochemical quality. Ind. Crops Prod..

[7] Shi X., Yang Y., Li Z., Wang X., Liu Y. (2020). Moisture transfer and microstructure change of banana slices during contact ultrasound strengthened far-infrared radiation drying. Innov. Food Sci. Emerg. Technol..

[8] Kahraman O., Malvandi A., Vargas L., Feng H. (2021). Drying characteristics and quality attributes of apple slices dried by a non-thermal ultrasonic contact drying method. Ultrason. Sonochemistry.

[9] Ismail M., Özbek N.H., Göğüş F. (2024). Hot air-assisted radio frequency drying of orange slices: Drying behavior and product quality. J. Food Sci..

[10] Li W., An N., Yu Z., Li D., Wang L., Wang Y. (2024). Enhancing Okra Drying Quality and Efficiency Through Combined Freeze and Pulsed Spouted Microwave Vacuum Drying. Food Bioprocess Technol..

[11] Suo K., Feng Y., Zhang Y., Yang Z., Zhou C., Chen W., Shi L., Yan C. (2024). Comparative Evaluation of Quality Attributes of the Dried Cherry Blossom Subjected to Different Drying Techniques. Foods.

[12] Deng L., Xiong C., Sutar P.P., Mujumdar A.S., Pei Y., Yang X., Ji X., Zhang Q., Xiao H. (2022). An emerging pretreatment technology for reducing postharvest loss of vegetables—A case study of red pepper (*Capsicum annuum* L.) drying. Dry. Technol..

[13] Zhou X., Wang S. (2019). Recent developments in radio frequency drying of food and agricultural products: A review. Dry. Technol..

[14] Zhou X., Li R., Lyng J.G., Wang S., Wang S. (2018). Dielectric properties of kiwifruit associated with a combined radio frequency vacuum and osmotic drying. J. Food Eng..

[15] Huang Z., Chen L., Wang S. (2015). Computer simulation of radio frequency selective heating of insects in soybeans. Int. J. Heat Mass Transf..

[16] Mao Y., Wang S. (2023). Recent developments in radio frequency drying for food and agricultural products using a multi-stage strategy: A review. Crit. Rev. Food Sci. Nutr..

[17] Luo X., Zhou D., Yang G., Xu J., Luo Y., Li R., Wang S. (2024). Development of radio frequency drying protocols for preserving the color and fragrance of lotus bee pollen. Food Control.

[18] Mahmood N., Liu Y., Munir Z., Zhang Y., Niazi B.M.K. (2020). Effects of hot air assisted radio frequency drying on heating uniformity, drying characteristics and quality of paddy. LWT-Food Sci. Technol..

[19] Huang Z., Zhang B., Marra F., Wang S. (2016). Computational modelling of the impact of polystyrene containers on radio frequency heating uniformity improvement for dried soybeans. Innov. Food Sci. Emerg. Technol..

[20] Gou M., Gu Y., Li W., Zheng J., Jiang H. (2020). Physicochemical characteristics, antioxidant capacity and thermodynamic properties of purple-fleshed potatos dried by radio frequency energy. Dry. Technol..

[21] Li F., Feng K., Yang J., He Y., Guo H., Wang S., Gan R., Wu D. (2020). Polysaccharides from dandelion (*Taraxacum mongolicum*) leaves: Insights into innovative drying techniques on their structural characteristics and biological activities. Int. J. Biol. Macromol..

[22] Zhang J., Li M., Cheng J., Wang J., Ding Z., Yuan X., Zhou S., Liu X. (2019). Effects of Moisture, Temperature, and Salt Content on the Dielectric Properties of Pecan Kernels during Microwave and Radio Frequency Drying Processes. Foods.

[23] Fernandes F.A.N., Rodrigues S. (2023). Ultrasound applications in drying of fruits from a sustainable development goals perspective. Ultrason. Sonochemistry.

[24] Xu B., Tiliwa E.S., Yan W., Azam S.M.R., Wei B., Zhou C., Ma H., Bhandari B. (2022). Recent development in high quality drying of fruits and vegetables assisted by ultrasound: A review. Food Res. Int..

[25] Gabriella D.d.S., Zilmar M.P.B., Rafael A.B.d.M., Carlos B.O.d.C., Shirley C.R.B., Patrícia M.A. (2016). Pretreatments for melon drying implementing ultrasound and vacuum. LWT-Food Sci. Technol..

[26] Pei Y., Li Z., Xu W., Song C., Li J., Song F. (2021). Effects of ultrasound pretreatment followed by far-infrared drying on physicochemical properties, antioxidant activity and aroma compounds of saffron (*Crocus sativus* L.). Food Biosci..

[27] Xi H., Liu Y., Guo L., Hu R. (2019). Effect of ultrasonic power on drying process and quality properties of far-infrared radiation drying on potato slices. Food Sci. Biotechnol..

[28] Liu Y., Zeng Y., Hu X., Sun X. (2020). Effect of ultrasonic power on water removal kinetics and moisture migration of kiwifruit slices during contact ultrasound intensified heat pump drying. Food Bioprocess Technol..

[29] Schössler K., Thomas T., Knorr D. (2012). Modification of cell structure and mass transfer in potato tissue by contact ultrasound. Food Res. Int..

[30] Shewale S.R., Hebbar H.U. (2017). Effect of infrared pretreatment on low-humidity air drying of apple slices. Dry. Technol..

[31] Bal L.M., Kar A., Satya S., Naik S.N. (2011). Kinetics of colour change of bamboo shoot slices during microwave drying. Int. J. Food Sci. Technol..

[32] Zang Z., Huang X., Zhang Q., Jiang C., Wang T., Shang J., He C., Wan F. (2023). Evaluation of the effect of ultrasonic pretreatment on vacuum far-infrared drying characteristics and quality of Angelica sinensis based on entropy weight-coefficient of variation method. J. Food Sci..

[33] Zhang Q., Wang T. (2017). Effect of ultrasound irradiation on the evolution of color properties and major phenolic compounds in wine during storage. Food Chem..

[34] Hao Q., Qiao X., Zheng Z., Lu X. (2021). Effects of ultrahigh pressure and ultrasound pretreatments on hot-air drying process and quality of garlic slices. Trans. Chin. Soc. Agric. Eng..

